# Virtual FBGs Using Saturable Absorbers for Sensing with Fiber Lasers

**DOI:** 10.3390/s18113593

**Published:** 2018-10-23

**Authors:** Luis Rodriguez-Cobo, Rosa A. Perez-Herrera, María A. Quintela, Rubén Ruiz-Lombera, Manuel Lopez-Amo, José M. Lopez-Higuera

**Affiliations:** 1 CIBER-bbn, Instituto de Salud Carlos III, 28029 Madrid, Spain; luis.rodriguez@unican.es (L.R.-C.); miguel.lopezhiguera@unican.es (J.M.L.-H.); 2 Department of Electrical Electronic and Communication Engineering and Institute of Smart Cities (ISC), Public University of Navarra, 31006 Pamplona, Spain; mla@unavarra.es; 3 Photonics Engineering Group, University of Cantabria, 39005 Santander, Spain; angeles.quintela@unican.es (M.A.Q.); ruben.ruiz@unican.es (R.R.-L.); 4 Instituto de Investigación Sanitaria Valdecilla (IDIVAL), 39005 Cantabria, Spain

**Keywords:** fiber bragg gratings, erbium doped fibers, fiber lasers, single-mode laser, temperature sensor, fiber optics sensors

## Abstract

The spectral narrowing of Fiber Bragg Gratings (FBGs) introduced by unpumped Er-doped fiber (EDF) was analyzed for fiber lasers (FL). Owing to spatial hole burning (SHB), the spectral response of a virtual FBG can be employed for narrowing the band pass filter employed to determine the lasing wavelength of laser cavities. A common FL was mounted to analyze the spectral stability of the method, and a FL sensor for strain and temperature measurements was experimentally characterized to determine the stability of the narrowing effect achieved by the unpumped EDF, which acts as a virtual FBG. The results exhibited remarkably good narrowing effects of the spectral response of uniform FBGs.

## 1. Introduction

Fiber lasers are important for practical engineering applications, such as: remote sensing, communications, health diagnosis, or structural monitoring [1]. In all of these scenarios, the quality of the light source may limit the final performance of the associated systems. 

Although most photonic systems take benefit from a light source insensitive to environmental parameters, in some scenarios, a source that depends on external perturbations can be useful as a sensing element by obtaining high precision measurements far from a host structure [2,3]. Both requirements (high precision and long distances) can be satisfied by fiber laser sensors, where one or several wavelengths matched FBGs can be employed to create an in-fiber cavity, where its output is a laser signal. There are many applications for FBG based lasers; however, when configured as sensing elements, changes in the environmental conditions (that affect the laser cavity, the FBGs, or both) can be detected by monitoring the laser output. 

Particularly, erbium doped fiber lasers (EDFL) are a very common structure to achieve high optical signal to noise ratio (OSNR) and a narrow linewidth, so they are suitable for their application in long-distance sensor systems [4,5]. However, EDFL also require a feedback stage that determines the oscillation wavelength, thus some filtering element should be employed, such as Fiber Bragg Gratings (FBGs). Fiber laser sensors based on FBGs usually exhibit the same linear response as passive gratings, but with some improvements, being their higher signal level as one of its most attractive features.

However, a number of drawbacks present in these lasers must be overcome before their widespread application. EDFL inevitably present oscillation in multiple longitudinal modes around its central wavelength because they usually have a large cavity length, a scenario that is even worse for ring cavities. Furthermore, it has to be noted that erbium-doped fiber (EDF) has a dominant homogeneous gain broadening behavior at room temperature, which means that the gain provided by the erbium at a specific wavelength will determine the gain at other wavelengths [6]. This implies that there is strong mode competition and usually suffers from unstable lasing. When applying EDFL to interrogate optical sensors, this inherent behavior of the EDFL is the major drawback for practical applications. 

Different techniques have been proposed to try to mitigate these drawbacks, being the most direct to achieve a single longitudinal mode (SLM) behavior. Typically, these techniques increase both system complexity and cost, which makes them unsuitable for many scenarios. For instance, the use of cavities with multiple-rings requires adjusting the length of the cavity and lasing parameters [7]. On the other hand, short cavity lasers have also demonstrated to attain SLM behavior [8]. In self-injection feedback lasers, the SLM behavior is only guaranteed if there are two channels and the power level is similar in both [9]. Other techniques consist of adding an ultra-narrow band width phase-shifted Fiber Bragg Grating (FBG) into the laser cavity, but the use of two combined gratings in the cavity introduces complexity when using this laser as a sensor [10].

Based on previous work presented at OFS25 [11], a stable SLM fiber ring laser for sensing purposes with a very simple configuration was experimentally evaluated. In this structure, a piece of unpumped EDF that acts as saturable absorber was introduced to achieve single-mode behavior [12]. Saturable absorbers have been successfully employed for narrowing different laser configurations [13,14]. In this work it has been experimentally demonstrated how, with a proper sizing of an EDF, the standing wave achieved between the laser cavity and its reflection in the FBG employed to select the lasing wavelength, may act as a very weak and long FBG that can modify the spectral response of the seed FBG that also can narrow the laser output. Moreover, different commercial Er-doped fibers were evaluated to create virtual FBGs and show its influence on the final laser properties. The best tested combination was also evaluated as strain and temperature sensors exhibiting a great linearity and stability.

## 2. Working Principles

In a simplified way, any fiber laser structure requires two key elements: An active medium to provide the amplification and an optical cavity that causes the positive feedback. Both elements, the active medium and the cavity design, determine the output power and the spectral laser properties. A common technique to select the lasing wavelength of a fiber ring laser is to use an FBG in reflection as feedback. In this way, the wavelength of interest is launched back into the amplification stage, and with the proper conditions, the lasing process starts. Since all the process takes place within an active medium, any slight variation of the feedback signal may have a remarkable influence on the achieved output. For example, in a ring cavity based on Er-doped fiber (EDF), slight variations in the reflection spectrum of an FBG are magnified by the gain competition proper of EDF [15], which usually makes the active spectral response of the system narrower than the passive FBG spectrum.

**Figure 1:**
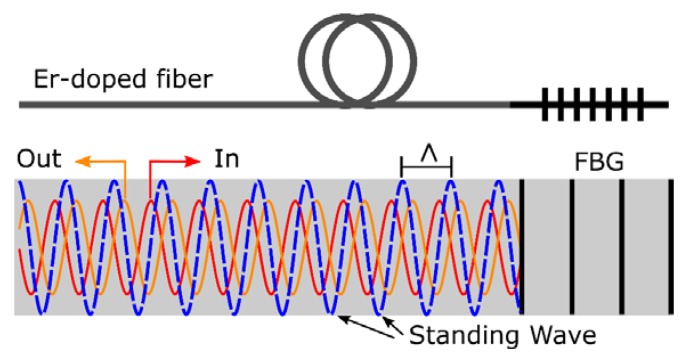
Schematic description of Fiber Bragg Gratings (FBG) induced by Spatial Hole Burning (SHB) with stationary wave on Er-doped fibers. Both incoming and outgoing waves create a standing wave that makes a periodic variation of the refraction index whose period matches the seed FBG.

Although there are many ways to modify the spectrum of an FBG, introducing an unpumped EDF before an FBG may also modify its spectral response [16]. When this combination is employed as feedback element into a fiber cavity, it may modify its spectral response, besides the attenuation introduced by the EDF absorption. Figure 1 shows a schematic description of FBG induced by spatial hole burning (SBH) with stationary wave on EDFs. 

When the same signal is forced to travel in both directions within an unpumped EDF, they create a standing wave, being the spatial period of light intensity distribution [12]:

(1) $$\Lambda =\frac{\lambda }{2n_{eff}},\;$$

*λ* being the central wavelength of the counter-propagating signal. This intensity modulation affects the absorption coefficient of the unpumped EDF (spatial-hole-burning), exhibiting a modulation of the same period ($\Lambda =\lambda /\left(2n_{eff}\right)$, which also provokes a periodical variation of the refraction index given by the Kramers-Kronig relation [17]. Assuming that similar power is available in both ways, a small periodical variation of the refraction index is present in the umpumped EDF, which can be understood as a very long weak virtual FBG, where its bandwidth is much smaller than the original FBG. Since the achieved index variation $\left(\bar{\Delta n_{eff}}\right)$ is very small (weak-grating limit), the bandwidth for the equivalent FBG can be approximated as in Reference [18]:

(2) $$\frac{\lambda_{0}}{\lambda }\propto \frac{\lambda_{d}}{n_{eff}L_{EDF}}=\frac{2}{N},\;$$

*N* being the total number of periods of the FBG induced into the unpumped Er-doped fiber. However, since the achieved index modulation is very weak, the achieved reflectivity of the induced grating will be also very low. It can be also approximated as in Reference [18]:

(3) $$R_{\max}=\tanh^{2}kL_{EDF},\;$$

where the coupling coefficient of the induced grating *k* is related to the weak index modulation, as in Reference [18]:

(4) $$k\propto \frac{\bar{\Delta n_{eff}}}{\lambda }.\;$$

Depending on several parameters, such as dopant concentration of EDF and power launched to the saturable absorber, the induced FBG properties will vary. When the induced index variation becomes higher, its achieved reflectivity will be enough to compensate the losses caused by the absorption, thus the equivalent FBG length (*L_EDF_*) will be higher. However, both index variation and absorption losses are related to the doping concentration, thus, each Er-doped fiber will present different optimum conditions to take advantage of this phenomenon.

**Figure 2:**
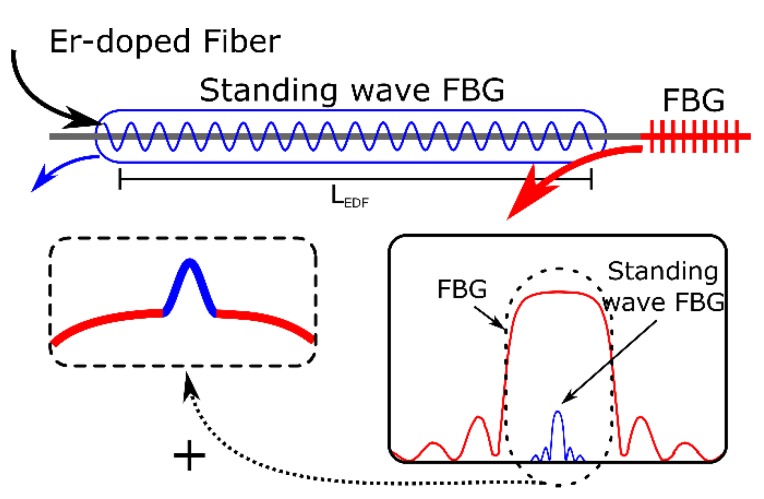
The FBG induced by the standing wave in the unpumped Er-doped fiber (EDF) modifies the passive response of the mirror FBG. Part of the incoming light is reflected by the induced FBG and dominates the active medium lasing properties due to gain competition of EDFs.

Understanding the standing wave FBG induced in the unpumped Er-doped fiber, as a virtual and very weak grating centered on the same wavelength of the passive FBG that slightly modifies its passive response, the problem can be modeled as a simple FBG combination. In Figure 2, an illustration of this approach has been depicted. The standing wave created by the incoming light and its reflection in the passive FBG can form a very long FBG with a very low reflectivity. Depending on the absorption on the unpumped EDF, this has an influence both in reflectivity of the induced FBG and in fiber losses, the equivalent FBG length (*L_EDF_*) will be modified, offering different reflection spectra. Moreover, the RI modulation created within the unpumped Er-doped fiber also produces Spectral Hole Burning, provoking that the fiber losses are dependent on the wavelength, meaning that slightly offset wavelengths would also see more absorption, favoring the filtering of the whole structure. Particularly, when a tradeoff between induced FBG Spectral Hole Burning and losses are achieved, the passive FBG spectral response is complemented with a very narrow FBG produced by the initial reflections on the standing wave FBG. Owing to the gain competition exhibited by Er-doped fibers, these slight differences produce very different laser signals.

This approach has been employed to build a fiber laser sensor, wherein we have tested different commercial Er-doped Fibers and their performance on narrowing the spectral response of the laser.

## 3. Experiments

**Figure 3:**
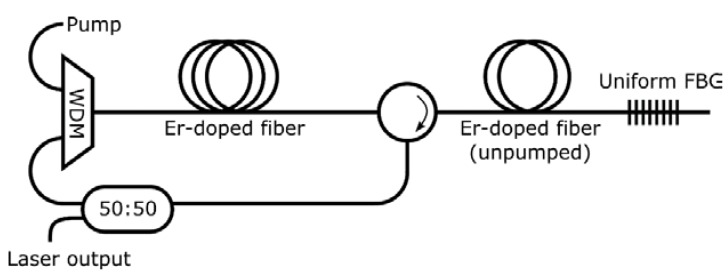
Erbium doped fiber sigma-cavity laser (EDSCL) sensor configuration. Two types of unpumped EDF block are introduced within the ring cavity before the FBG to evaluate the active lasing modes.

An erbium doped fiber sigma-cavity laser (EDSCL) was employed to analyze the performance of this configuration when different kinds of saturable absorbers are included into the sigma-cavity, as Figure 3 shows. A commercial EDF (4 m of I25 of Fibercore) was employed as active medium within the cavity, while it was pumped with a 980 nm laser for both experiments. A uniform FBG centered on 1552 nm, as well as a circulator, were used as feedback element to launch the desired wavelengths into the ring cavity. This configuration was analyzed by introducing two types of unpumped EDF block with the same length (2 m of I25 and 2 m of M12), introducing them after the circulator. To remark the importance of the doping density of the EDF used in the saturable absorber, two different types of unpumped EDFs were analyzed. I25 showed an absorption of 24.2 dB/m, meanwhile, M12 had an absorption of 12.71 dB/m both at 979 nm. These different absorptions lead to different optical behaviors of the laser.

Optical fiber circulator generates the phase delay needed to avoid a phase-shift of 0 radians when light reaches the grating, and it also attenuates the remaining pump power, so that Er-doped fiber located after the circulator does not amplify the received signal and instead acts as a saturable absorber.

Previous works have demonstrated that when no unpumped EDF is employed, the resulting laser works on a multimode regime [11]. In addition to this, when the unpumped fiber is included into the cavity the same happens, where the laser signal only travels in one direction. Adding a saturable absorber into the cavity helps to reduce the gain competition on the active fiber, so the obtained spectral output shows less modes. On the other hand, when placing the EDF just before the FBG, light travels in both directions achieving the narrowing effect explained in Reference [11], and outputting a laser emission working on the SLM regime. A further characterization of these properties was performed.

### 3.1. Spectral Characterization

**Figure 4:**
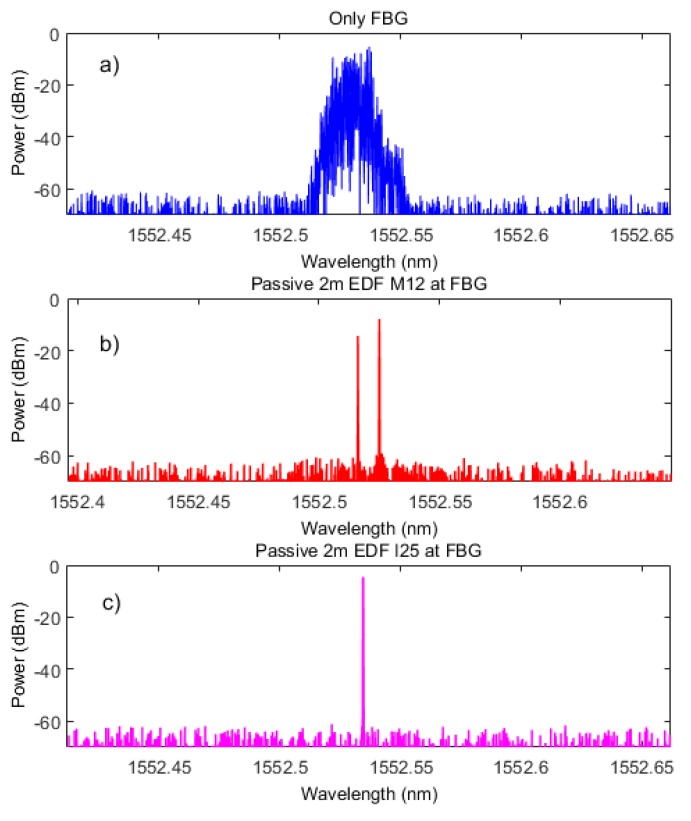
High-resolution output spectra of the EDSCL measured by a BOSA of each tested configuration: no passive EDF (**a**), 2 m of EDF M12 (**b**), or 2 m of EDF I25 (**c**) as passive element.

To evaluate the active lasing modes, a high-resolution optical spectrum analyzer (BOSA-C of Aragon Photonics) which offers high resolution (0.08 pm) and high dynamic range (>80 dB) simultaneously, was used. These three different configurations were measured, while the pump power was maintained at 100 mW at 980 nm. As depicted in Figure 4, when no EDF is included into the laser, the laser outputs a number of active modes (Figure 4a).

When a passive EDF is placed before the FBG, the number of active modes is drastically reduced due to the narrowing effect provoked by the saturable absorber (unpumped EDF). That can be observed in Figure 4b,c, where 2 m of EDF of M12 or I25 were used, in that order.

When no EDF is placed, the laser output is wider, exhibiting different lasing modes. However, when a short unpumped EDF is introduced, the number of lasing modes decreases, even when the tradeoff between the induced grating reflectivity and the attenuation due to absorption is not in its optimum value, such as happens with the low concentration EDF (M12 from Fibercore). The required tradeoff was achieved with 2 m of I25 EDF of Fibercore, resulting in a very stable fiber ring laser working on the SLM regime.

**Figure 5:**
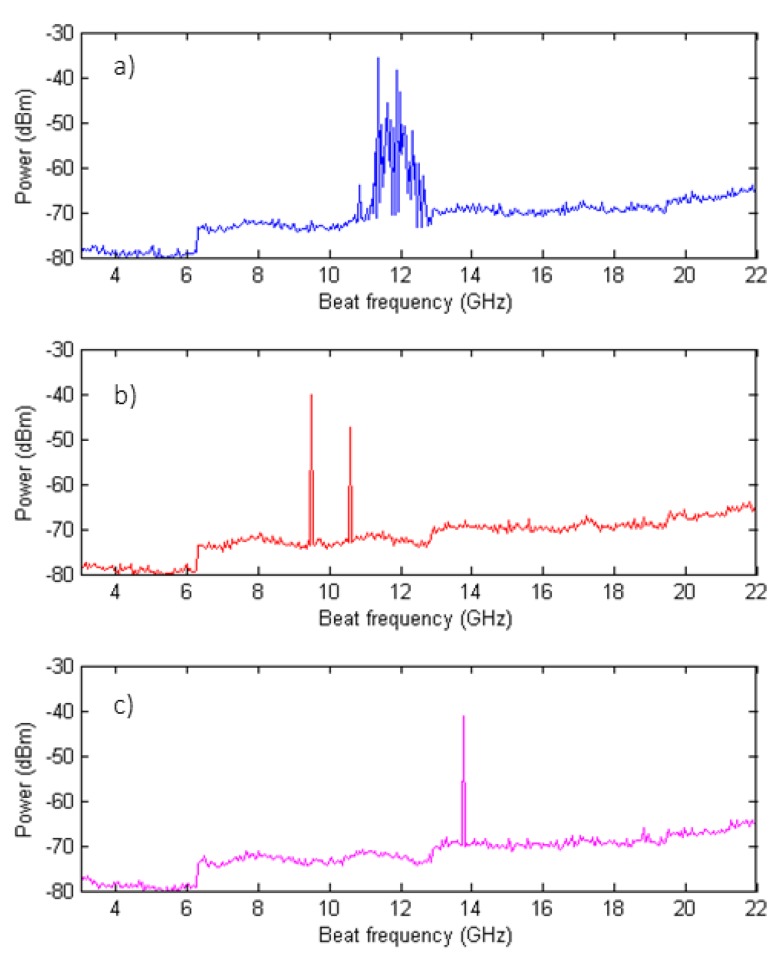
Electric beat with a tunable laser source (TLS) of the laser output for each tested configuration: No unpumped EDF (**a**), 2 m of unpumped EDF M12 (**b**), or 2 m of unpumped EDF I25 (**c**). SLM can be reached with an unpumped EDF and a common FBG as filter elements on a fiber laser.

To verify the number of lasing modes of the fiber ring laser with the different configurations of unpumped EDF, the laser output was mixed with the signal of a tunable laser source (TLS) using a 3 dB coupler to perform the heterodyne detection. The TLS (Agilent 8164B) had a full-width at half maximum (FWHM) linewidth of 100 kHz, and its wavelength was placed close to the manufactured laser. The achieved results are depicted in Figure 5, and confirm the measures obtained with the high resolution optical spectrum analyzer (BOSA-C).

**Figure 6:**
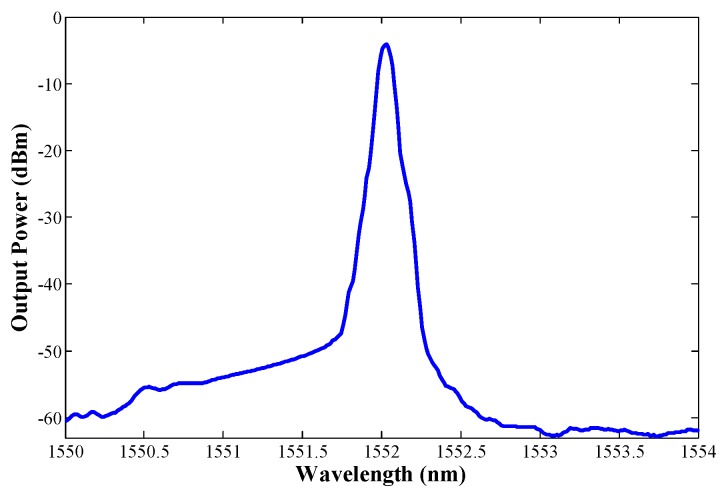
Output spectra measured by the OSA when using 2 m of EDF M12.

Figure 6 depicts the output spectrum of this EDSCL when 2 m of M12 were used and measured by an OSA. The output power obtained was very similar for both configurations, using M12 or I25 as unpumped EDF. An output power level of around −3.5 dBm and an optical signal to noise ratio (OSNR) of about 40 dB were measured in both cases. Several previous studies show that these values are reasonably good for most sensor applications [19].

### 3.2. Wavelength and Power Stability

**Figure 7:**
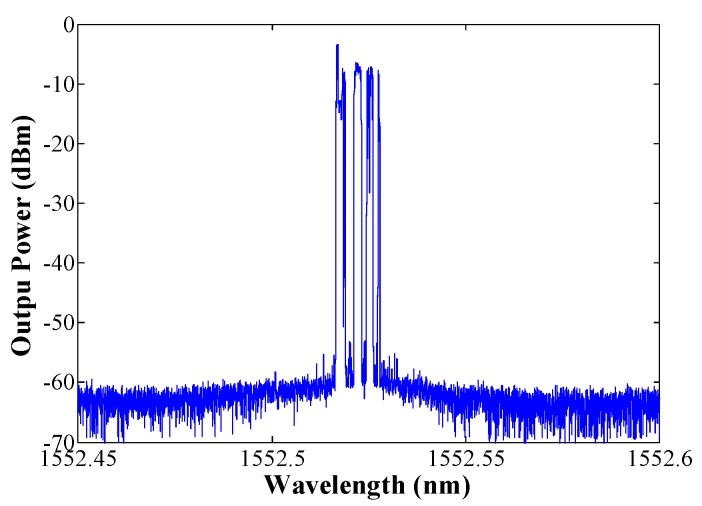
Hold trace of the laser emission. It exhibits a wavelength variation under 11 pm over 10 min when 2 m of M12 was used as unpumped EDF.

Maintaining the pump power constant (100 mW at 980 nm) and using 2 m of EDF M12, the output wavelength was also evaluated. The measured output wavelength variation over 10 min was as low as 11 pm, as can be observed in the hold trace depicted in Figure 7.

**Figure 8:**
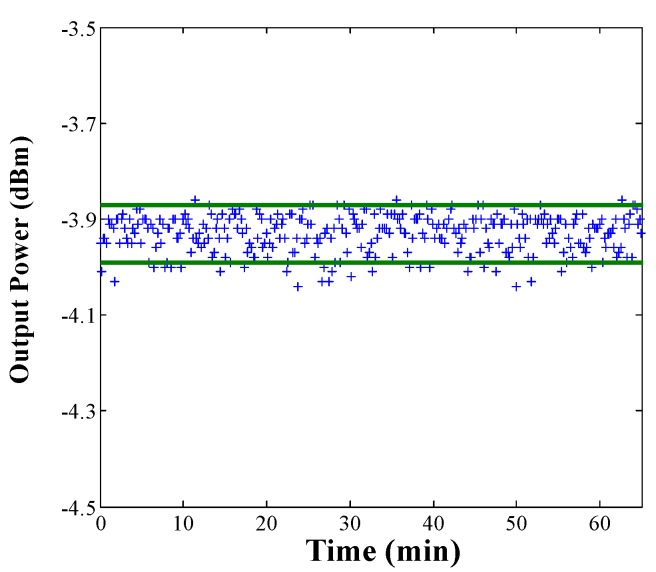
Output power stability measured for 60 min with constant pump power (100 mW) when 2 m of EDF I25 was used.

The output power stability measured each 30 s is depicted in Figure 8, achieving a drift of 0.12 dB with a 90% of confidence level (CL) when 2 m of EDF I25 was employed, exhibiting an improvement in terms of stability when compared with previous works [15].

### 3.3. Sensor Response

**Figure 9:**
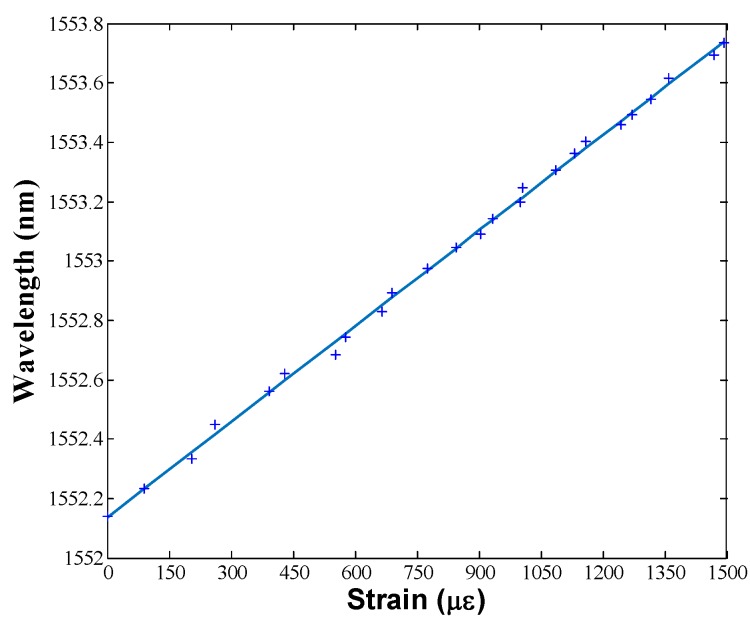
Strain sensitivity of the EDSCL. The whole erbium doped fiber ring laser (EDFRL) sensor exhibits the same sensitivity as the FBG employed to select the lasing wavelength.

After wavelength and spectral characterization of these configurations, the attained fiber laser by employing the EDF I25 was tested for sensor measurements. Strain and temperature variations were applied over the uniform FBG. A micrometric linear motor stage was used to perform a strain sweep producing a peak variation of around 1500 µε at room temperature (25 °C). A linear response, with a strain sensitivity of 1.06 pm/µε, were obtained as Figure 9 shows. 

**Figure 10:**
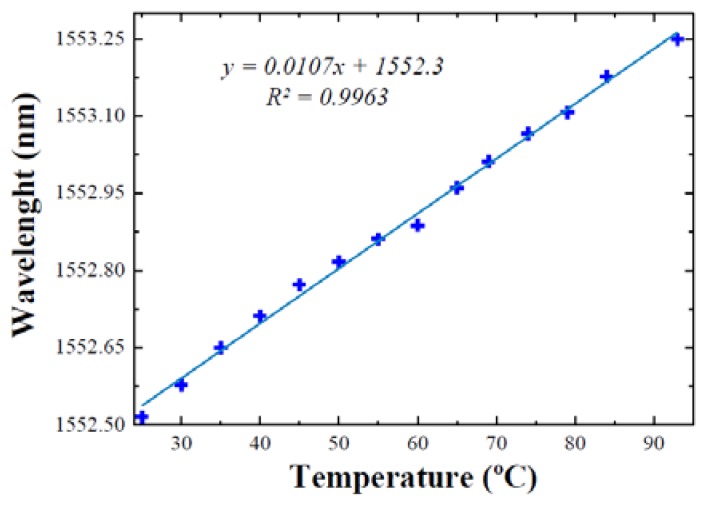
Temperature sensitivity of the EDSCL. The whole EDSCL sensor exhibits the same sensitivity as the FBG employed to select the lasing wavelength.

The temperature response of the FBGs as temperature sensor was also tested by heating it on a climatic chamber in the range of 25 °C to 95 °C. As can be seen in Figure 10, the center wavelength shift for the laser centered at 1552 nm presents a clear linear behavior and a temperature sensitivity of about 10.7 pm/°C was measured. These measurements were also carried out by using the 2 m of EDF M12, obtaining very similar results, and demonstrating the viability of this configuration to be used as a sensor system.

## 4. Conclusions

In this work, it was experimentally demonstrated how a standing wave can be achieved between a laser cavity and its reflection in the FBG employed to select the lasing wavelength. With a proper sizing of the EDF, this standing wave may act as a very weak and long FBG that can modify the spectral response of the seed FBG. This reduces the bandwidth of the emitted laser line, reaching the SLM regime. In this manner, the peak tracking of the virtual-FBG-based sensor is easier and the resolution is increased. Based on commercial EDFs, a typical fiber laser has been employed to evaluate the influence the induced FBG created into an unpumped EDF before the mirror FBG. This setup was also tested for sensing both strain and temperature and exhibited the linearity and repeatability expected of a typical FBG. During all these tests, the spectral properties of the laser were maintained, proving the stability of the proposed scheme.
